# Prognostic significances of overexpression MYC and/or BCL2 in R-CHOP-treated diffuse large B-cell lymphoma: A Systematic review and meta-analysis

**DOI:** 10.1038/s41598-018-24631-5

**Published:** 2018-04-19

**Authors:** Lu Li, Yanyan Li, Ximei Que, Xue Gao, Qian Gao, Mingxing Yu, Kaili Ma, Yanfeng Xi, Tong Wang

**Affiliations:** 1 Department of Health Statistics, School of Public Health, Shanxi Medical University, Taiyuan, 030001 China; 2 Department of Pathology, Shanxi Tumor Hospital, Taiyuan, 030013 China

## Abstract

Numerous studies have investigated the prognostic values of MYC and/or BCL2 protein overexpression in diffuse large B-cell lymphoma (DLBCL). However, the results still demonstrate discrepancies among different studies. We aimed to do a systematic review and meta-analysis on the relationships between overexpression MYC and/or BCL2 and DLBCLs treated with rituximab, cyclophosphamide, doxorubicin, vincristine, and prednisone (R-CHOP). This study followed the guidelines of PRISMA and Cochrane handbook. The hazard ratios (HRs) for overall survival (OS) were pooled to estimate the main effect size. Twenty studies recruited a total of 5576 patients were available for this meta-analysis. The results showed that MYC (HR = 1.96, 95%CI (confidence interval) = 1.69–2.27)without heterogeneity(I^2^ = 17.2%, P = 0.280), BCL2 (HR = 1.65, 95%CI = 1.43–1.89, I^2^ = 20.7%, P = 0.234) protein overexpression, and co-overexpression (HR = 2.58, 95%CI = 2.19–3.04, I^2^ = 17.2%, P = 0.275) had a poor prognosis in R-CHOP treated DLBCL patients, respectively. The current analysis indicated that MYC and/or BCL2 protein overexpression, and particularly co-overexpression was related to short overall survival in R-CHOP treated DLBCL patients, showing that application of the two new biomarkers can help to better stratify DLBCL patients and guide targeted treatment.

## Introduction

Diffuse large B-cell lymphoma (DLBCL) is the most common subtype of aggressive non-Hodgkin lymphoma (NHL) and a highly heterogeneous malignancy of B cells both biologically and clinically^1^. The International Prognostic Index (IPI) system has been widely used to predict prognosis in patients with high grade NHL^2^. However, there still remain obvious distinctions in clinical outcomes within the high risk subgroup, suggesting other potential contributing factors that IPI couldn’t explain^3^. Meanwhile, DLBCL is remediable in more than 60% patients when treated with standard treatment, known as R-CHOP (rituximab, cyclophosphamide, doxorubicin, vincristine, and prednisone)^4^, so it is important to seek out optimal biomarkers which could identify the rest of patients who failed to be cured with R-CHOP treatment.

Diverse genetics and proteomics studies have been explored in DLBCL^5,6^. Earlier studies reported the poor prognosis of MYC and BCL2 and/or BCL6 rearrangements in DLBCL, known as double-hit lymphoma (DHL), or triple hit lymphoma (THL) by using fluorescent *in situ* hybridization (FISH) cytogenetic techniques. In 2016, the World Health Organization (WHO) revision of the lymphoma classification defined this as a new category of high-grade B-cell lymphoma (HGBL)^7^. Additionally, this new type of HGBL occurred in less than 10% DLBCL patients. In comparison, the co-overexpression of MYC and BCL2 and/or BCL6 proteins, so-called dual expressors (DE-DLBCL) or triple expressors(TE-DLBCL) detected by Immunohistochemistry(IHC) is much more common, occurring in 20–30% DLBCL patients. This higher percentage scope may make detailed subdivisions of patients. Moreover, FISH fails to detect gene deregulation caused by further mechanisms other than translocation level, but protein is a more effective molecule that can make up for the above deficiency and control physiological function directly. Over the last five years, the assessment of overexpression MYC and BCL2/BCL6 has emerged as frequently-used biomarkers for prognosis in DLBCL patients. However, there are many controversial issues about diagnosis, treatment, and prognosis in DE-DLBCL patients, including acknowledged cut-off values for each protein overexpression, uniform therapy regimens, final outcomes, and so on. Therefore, the poor prognostic implications of overexpression MYC and/or BCL2 still remain undetermined. Furthermore, there are far less data available for BCL6 protein expression, part of the reason is its rarity. So the systematic review and meta-analysis aims to illuminate the prognostic values of MYC and/or BCL2 overexpression in R-CHOP-treated DLBCL patients.

## Materials and Methods

This study followed the guidelines of the Meta-analysis of Observational Studies in Epidemiology group (MOOSE)^8^ and Preferred Reporting Items for Systematic Reviews and Meta-analysis (PRISMA)^9^.

### Search strategy and selection criteria

We performed a literature search in PubMed, Embase and Cochrane Library to identify all primary research studies which evaluate the associations between MYC and/or BCL2 overexpression and prognosis in DLBCLs. The electronic search was performed combining Medical Subject Headings (MeSH) and text words, using the following terms: “MYC”, “BCL2”, “Lymphoma, Large B-Cell, Diffuse/DLBCL” and “prognosis/prognostic/survival”. The language was restricted to English. All the studies published before 24 October 2017 were included. We also retrieved additional articles through references included in the eligible studies and relevant reviews.

The following included criteria were established: (1) all included patients should be pathologically confirmed in diagnosis of DLBCL according to the World Health Organization classification of tumors of the hematopoietic and lymphoid tissues; (2) sufficient information about MYC and/or BCL2 protein overexpression levels should be provided; (3) the association between MYC and/or BCL2 protein overexpression and DLBCL prognosis should be reported. Articles were excluded if they (1) were case reports, letters, commentaries, meeting records or review articles; (2) included patients with human immunodeficiency virus infection(HIV) infection, epstein-barr virus(EBV) infection or primary central nervous system disease; (3) lacked sufficient data for estimating hazard ratios (HRs) and their 95%CIs(confidence intervals). Additionally, if more than one study by the same author using the same case series were published, either the study with the larger sample size or the most recently published was selected.

### Quality assessment

Two researchers (Lu Li and Ximei Que) independently assessed the study quality according to the Newcastle-Ottawa Scale (NOS) for cohort and case-control studies^10^. Any disagreements were resolved by rigorous discussions. The NOS criteria included the following three aspects: (1) Selection: 0–4; (2) Comparability: 0–2; (3) Outcome/Exposure: 0–3. A study can be awarded a maximum of one score for each numbered item within the Selection and Exposure categories. A maximum of two scores can be given for Comparability. NOS scores range from 0 to 9 with no less than 6 indicates good quality.

### Data extraction

The extracted contents included the first author’s name, publication year, studying country, cut-off values of MYC and/or BCL2 protein expression, number of patients, sampling type, detection method, follow-up duration and HRs with 95% CIs for OS. If the HR was not reported directly, we can extract data from the survival curve published in the article and then estimated the HRs by using Engauge Digitizer version 4.1. All the calculations mentioned above were based on the methods introduced by Parmar^11^ and Tierney^12^. If necessary, we also contacted the corresponding author of included articles in order to obtain additional information.

### Statistical analysis

We pooled HRs of the studies by using Stata12.0 (version 12.0, Stata Corporation Station, TX). An observed HR > 1 indicated a worse prognosis for the group with relevant protein overexpression, whereas HR < 1 implied a better prognosis for the group with relevant protein overexpression. Besides, if the 95%CI of HR included the null value, that is 1, then this HR was not statistically significant. Heterogeneity among studies was evaluated using the Cochrane’s Q test and I^2^ index. A P < 0.1 for the Q-test or I^2^ > 50% indicated heterogeneity among the studies. The random-effects model was chosen to estimate the combined HR if heterogeneity was significant (P < 0.1, I^2^ > 50%). Otherwise, the fixed-effects model was used (P > 0.1, I^2^ < 50%). Subgroup analysis was implemented to explore sources of heterogeneity. Sensitivity analysis which used the leave-one-out method was performed to assess the outcome stability by evaluating the influence of singular study. The publication bias of included studies was assessed by visual inspection of funnel plots and two formal statistical tests for asymmetry of the funnel plots, including Begg’s rank correlation test^13^ and Egger’s linear regression test^14^. A symmetric graphic revealed unlikely publication bias.

## Results

### Characteristics of the included studies

**Figure 1:**
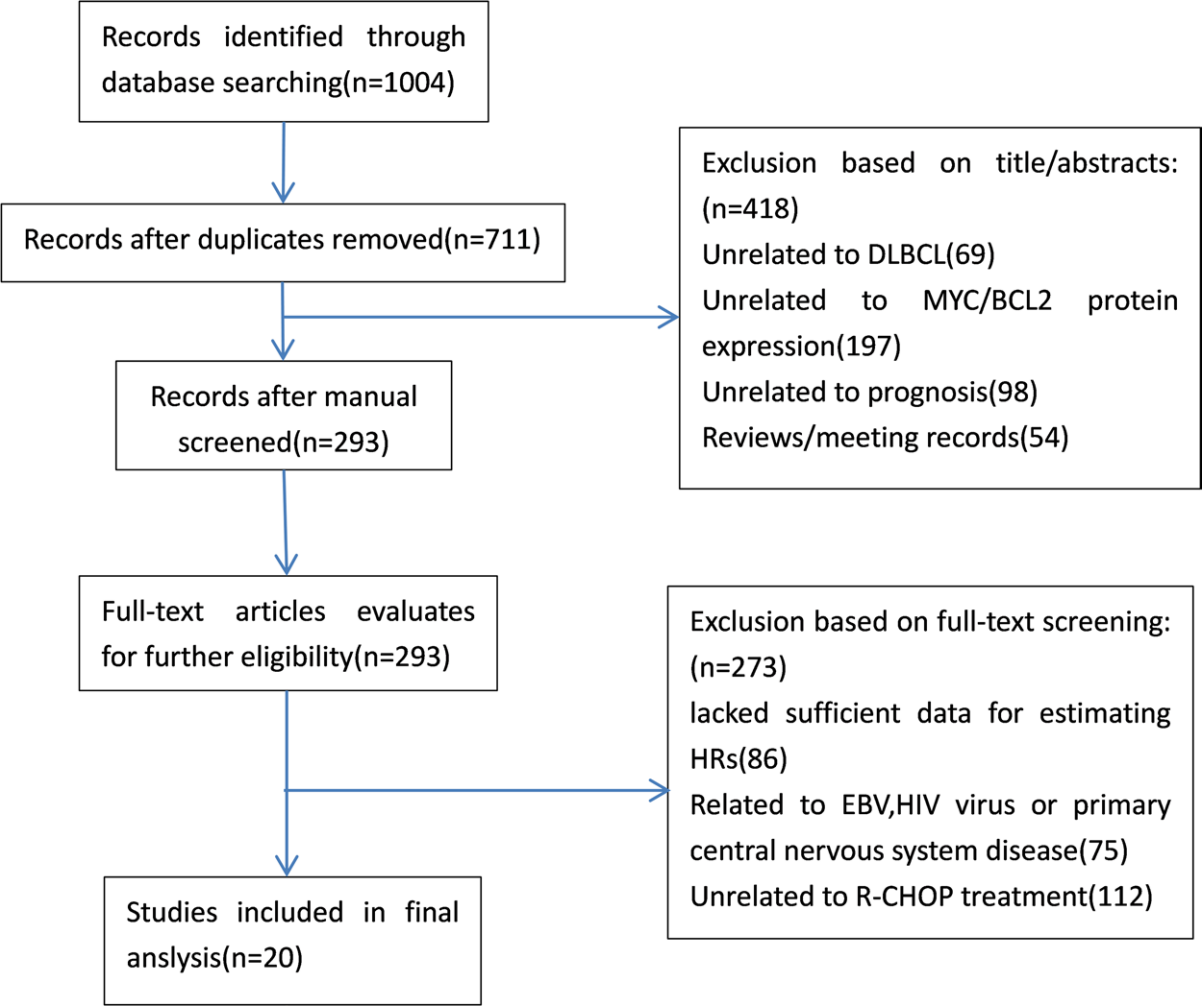
Flow chart of the study selection process.

According to the search strategy, a total of 1004 potentially relevant articles were identified in PubMed, Embase and Cochrane Library. After a browse of the titles and abstracts and then assessment of the full-text, twenty studies which included a total of 5576 enrolled patients were available for this meta-analysis. Figure 1 shows the selection process of these studies.

The involved studies were all published between 2008 and 2017. Most of the studies used the method of IHC to measure the gene expression, while only one study used the method of Array Plate quantitative nuclease protection assay (qNPA) technology. The type of specimen used in eighteen studies was formalin-fixed, paraffin-embedded tissue (FFPE), only two studies used tissues without further explanation. The HRs were extracted from fifteen studies which reported them directly and extrapolated from the survival curves in the other five studies which did not report HRs. These studies were retrospective excepted for a prospective one^15^, and all recorded the effect of MYC and/or BCL2 protein overexpression on OS. The details and methodological qualities of twenty studies are shown in Table 1.

**Table 1:** Features summary of the enrolled studies in the meta-analysis.

Study(year)	Region	Gene	Number of patients	Sample type	Cut-off value	Detection method	Median follow-up monthes (minimum,maximum)	HR(95%CL)	Outcome	Quality	Hazard ratio
Zhou(2014)^47^	China	MYC	60	FFPE	50%	IHC	24(4,62)	11.862(1.462,96.218)	OS	7	R
Zijun(2015)^48^	Western	BCL2	828	FFPE	70%	IHC	NR	2.04(1.45,2.87)	OS	7	R
Lisa(2008)^49^	America	MYC	116	FFPE	50%	qNPA	NR	1.64(1.16,2.31)	OS	7	R
	America	BCL2	116	FFPE	50%	qNPA	NR	1.11(0.77,1.60)	OS	7	R
Iqbal(2011)^50^	Mix	BCL2	221	TISSUE	50%	IHC	NR	2.0(1.0,4.0)	OS	7	R
Johnson(2012)(T)^18^	Mix	MYCBCL2	164	FFPE	40%50%	IHC	42(6.24,135.6)	4.27(2.18,8.37)	OS	8	SC
(V)	British	MYCBCL2	140	FFPE	40%50%	IHC	56.4(12.0,96.0)	1.47(0.75,2.87)	OS	8	SC
Ye(2015)^51^	America	MYC	825	TISSUE	70%	IHC	58.9(1,187)	1.89(1.26,3.94)	OS	8	R
	America	BCL2	849	TISSUE	70%	IHC	58.9(1,187)	1.67(1.14,2.46)	OS	8	R
	America	MYCBCL2	831	TISSUE	70%70%	IHC	58.9(1,187)	2.54(1.65,3.94)	OS	8	R
Scott(2015)^52^	British	MYCBCL2	339	FFPE	40%50%	IHC	78(9,158.4)	1.9(1.4,3.1)	OS	7	R
Fan(2015)^53^	China	MYC	141	FFPE	40%	IHC	30(3,112)	3.127(1.649,5.929)	OS	8	R
	China	BCL2	141	FFPE	50%	IHC	30(3,112)	0.934(0.465,1.875)	OS	8	R
Perry(2013)(T)^54^	America	MYC	106	FFPE	50%	IHC	NR	2.15(1.08,4.31)	OS	7	SC
	America	BCL2	106	FFPE	30%	IHC	NR	2.06(1.07,3.96)	OS	7	SC
	America	MYCBCL2	106	FFPE	50%30%	IHC	NR	9.24(1.2,70.64)	OS	7	R
(V)	British	MYCBCL2	205	FFPE	40%50%	IHC	NR	2.79(0.37,21.38)	OS	7	R
Green(2012)(T)^35^	Denmark	MYCBCL2	193	FFPE	40%70%	IHC	56(1,99)	4.48(2.69,7.44)	OS	8	R
(V)	Mix	MYCBCL2	116	FFPE	40%70%	IHC	33(1,102)	2.44(1.23,4.86)	OS	8	R
Hu(2013)^17^	Mix	MYC	466	FFPE	40%	IHC	57	1.77(1.26,2.48)	OS	8	SC
	Mix	BCL2	466	FFPE	70%	IHC	57	2.00(1.45,2.74)	OS	8	SC
	Mix	MYCBCL2	411	FFPE	40%70%	IHC	57	2.52(1.73,3.67)	OS	8	R
Yan(2014)^16^	China	MYC	118	FFPE	40%	IHC	37(1,145)	4.12(1.86,9.10)	OS	7	R
	China	BCL2	118	FFPE	70%	IHC	37(1,145)	1.48(0.71,3.07)	OS	7	R
	China	MYCBCL2	115	FFPE	40%70%	IHC	37(1,145)	2.67(1.60,4.48)	OS	7	R
Xu(2017)^55^	China	MYCBCL2	204	FFPE	40%70%	IHC	40.5(0.6,154.2)	2.384(1.222,4.652)	OS	8	R
Keisuke(2016)^56^	Japan	MYC	61	FFPE	30%	IHC	40(2,127)	1.361(0.556,3.334)	OS	8	R
	Japan	BCL2	61	FFPE	1%	IHC	40(2,127)	3.481(1.158,10.46)	OS	8	R
Kluk(2012)^57^	America	MYC	38	FFPE	50%	IHC	31(2,69)	5.22(0.35,77.47)	OS	7	SC
Horn(2013)^15^	Germany	MYC	135	FFPE	40%	IHC	NR	2.3(1.2,4.7)	OS	8	R
	Germany	BCL2	135	FFPE	1%	IHC	NR	4.5(1.3,16.2)	OS	8	R
Kendrick(2014)(I)^58^	America	BCL2	44	FFPE	50%	IHC	NR	0.983(0.285,3.395)	OS	8	R
(S)	America	BCL2	102	FFPE	50%	IHC	NR	1.18(0.52,2.67)	OS	8	SC
Kelli(2015)^59^	America	MYCBCL2	69	FFPE	40%50%	IHC	4.25(0.14,12.85)	2.63(1.07,6.44)	OS	8	R
Salles(2011)^60^	Mix	BCL2	326	FFPE	75%	IHC	4.4	1.4(0.9,2.2)	OS	8	R
Monette(2015)^20^	Mix	MYC	535	TISSUE	70%	IHC	45(30,176.1)	1.83(1.4,2.41)	OS	8	R

IHC: Immunohistochemistry FFPE: formalin-fixed, paraffin-embedded tissue R: reported qNPA: quantitative nuclease protection assay SC: survival curve NR: not reported T: training set V: validation set I: initial cohort S: second cohort TMA: tissue microarrays.

### Meta-analysis

**Figure 2:**
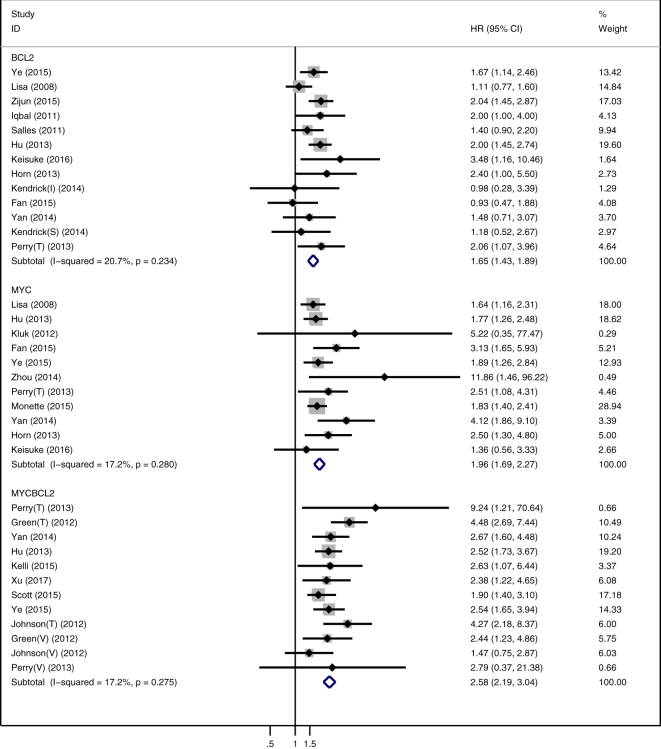
Forests plots of HRs for MYC and/or BCL2 protein expression. The point estimate is bounded by a gray box (its size is proportional to the study weight) and a horizontal line indicated the 95%CI, the vertical line represents no effect on the outcome and diamonds represent the pooled HRs.

No significant heterogeneity among the included studies was detected in this meta-analysis, and the fixed-effect model was used to assess the pooled HRs for OS. There were eleven articles related to the expression of MYC protein. The combined HR of MYC protein overexpression was 1.96 (95%CI, 1.69–2.27) without heterogeneity (I^2^ = 17.2%, P = 0.280). Thirteen articles reported the association between BCL2 protein expression and prognosis of DLBCL. The pooled HR was 1.65 (95%CI, 1.43–1.89, I^2^ = 20.7%, P = 0.234). Additionally, twelve articles related to MYC and BCL2 protein co-overexpression and the combined HR was 2.58 (95%CI, 2.19–3.04, I^2^ = 17.2%, P = 0.275). The above results showed that MYC and BCL2 protein overexpression alone or co-overexpression can lead to inferior outcome, and the prognostic significance of co-overexpression is more prominent. Detailed results of the meta-analysis for MYC and/or BCL2 protein overexpression are listed in forest plots (Fig. 2).

### Sensitivity analyses and publication bias

**Figure 3:**
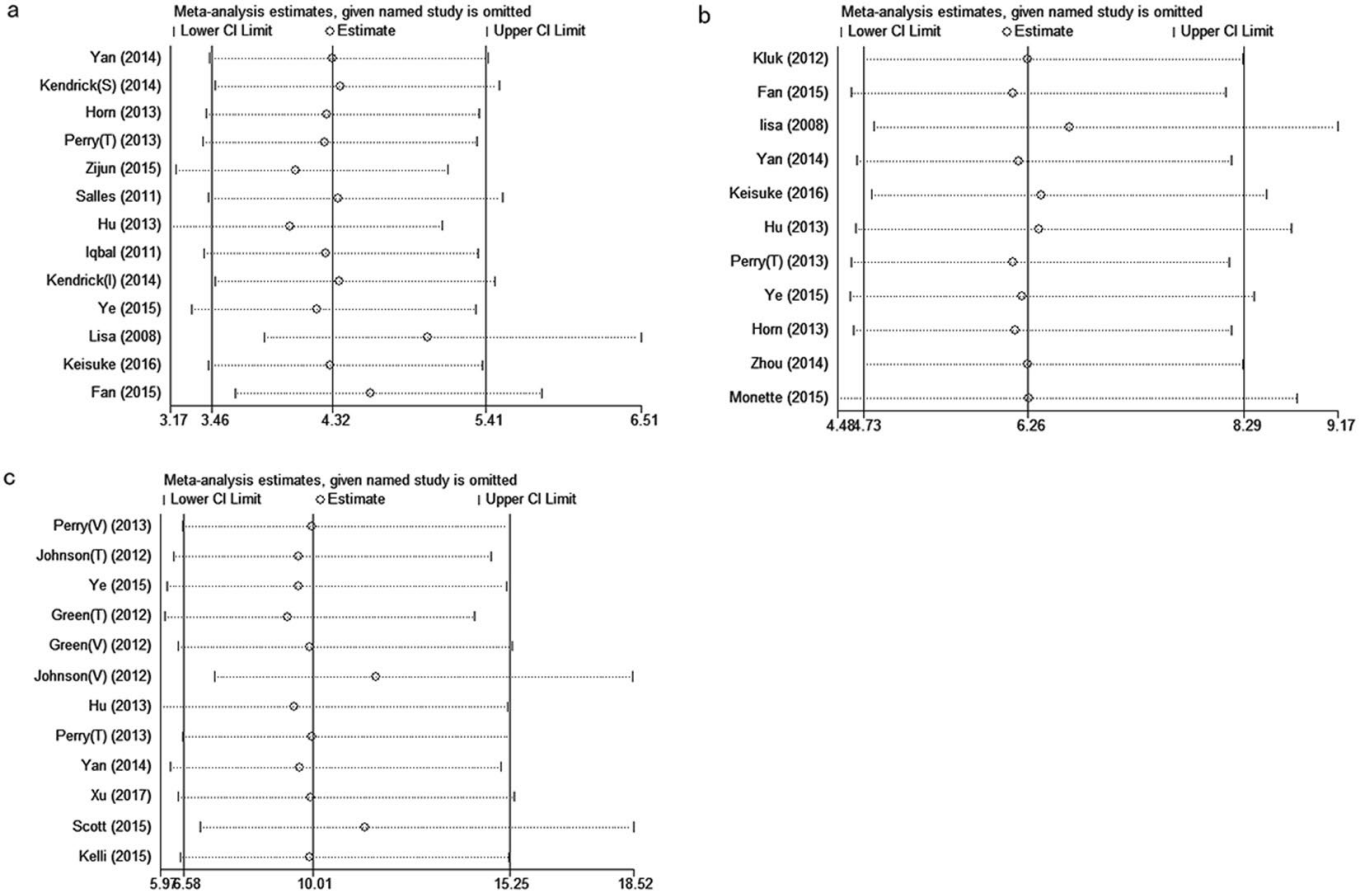
Sensitivity analysis for MYC and/or BCL2 overexpression. (**a**) BCL2 protein overexpression. (**b**) MYC protein overexpression. (**c**) MYC and BCL2 protein co-overexpression.

**Figure 4:**
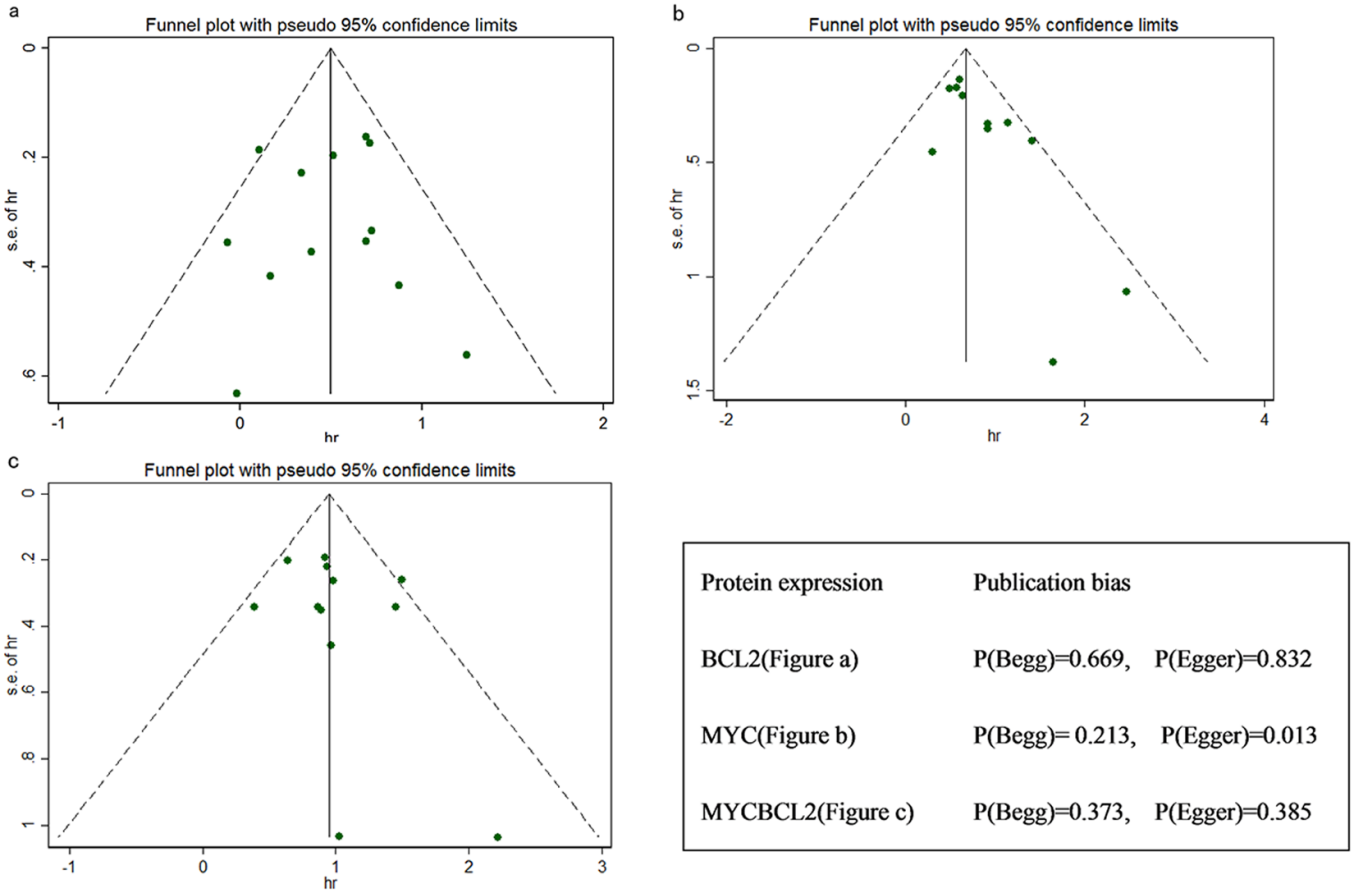
Funnel plots of publication bias and relevant Begg’s and Egger’s tests. (**a**) BCL2 protein overexpression. (**b**) MYC protein overexpression. (**c**) MYC and BCL2 protein co-overexpression.

The results of sensitivity analyses indicated that removal of any study could not affect the overall pooled HRs (Fig. 3). There was no significant evidence for asymmetry in the funnel plots, and no significant evidence of publication bias was presented by Begg’s and Egger’s tests, except for the MYC protein overexpression. In Fig. 4b, there were two studies located at the right bottom which influenced the symmetry of funnel plot and the result of Egger’s test, but Begg’s test showed that there is no sign of bias. However, when comparing Egger’s test and Begg’s test, the former is more sensitive and the latter more conservative. Meanwhile this phenomenon can also be interpreted in view of the small number of included articles (n = 11) (Fig. 4).

### Subgroup analyses

**Figure 5:**
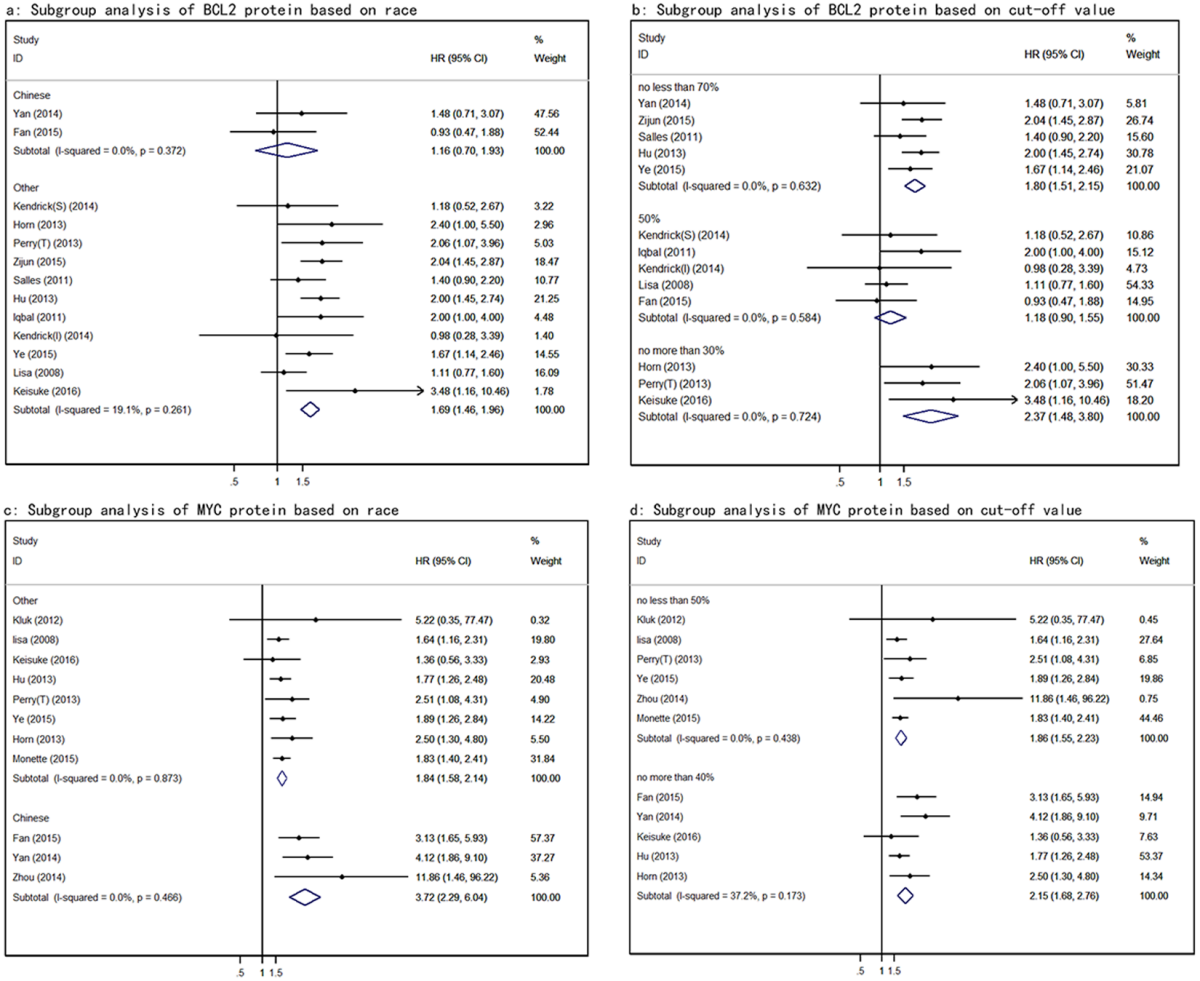
Subgroup analyses based on cut-off value and race. (**a**) subgroup analysis of BCL2 protein based on race. (**b**) subgroup analysis of BCL2 protein based on cut-off value. (**c**) subgroup analysis of MYC protein based on race. (**d**) subgroup analysis of MYC protein based on cut-off value.

Low degree of heterogeneity was observed in our analyses with all three I^2^ < 25%. Yan *et al*. presented that DLBCLs in China appears to have many characteristics different from those in Western counties^16^. On the other hand, the cutoff values in different studies were established through different analysis including receiver-operating characteristic curves^17^, X-Tile statistical software^18^, and other means to determine the appropriate points, that lead to inconsistency. Accordingly, we can see that possible sources of heterogeneity included race and cut-off value, so the subgroup analyses were performed in terms of the two aspects. The subgroup analysis indicated that the heterogeneity source of BCL2 protein overexpression comes from cutoff which reveals that 50% had no effect on prognosis. The causes of this phenomenon were that the lower and higher cutoff values have preferable true positive rate and true negative rate respectively. And in terms of subgroup analysis by race, the heterogeneity of MYC protein overexpression comes from different populations. Furthermore, we did not do a subgroup analysis of MYC and BCL2 co-overexpression given subgroups too much (Fig. 5).

## Discussion

Nowadays, protein overexpressions and gene rearrangements of MYC and BCL2 involved in “double-protein” and “double-hit” DLBCL are the most commonly used biomarkers to predict the poor prognosis in DLBCL patients treated with R-CHOP. “Double-hit” DLBCL is recognized by most as a less favorable prognostic factor. It is widely acknowledged that most institutions test MYC and BCL2 rearrangements for newly diagnostic DLBCLs, but protein overexpression is not routinely performed since there still has a small difference among studies. In addition, there are three reasons why we analyze protein expression: firstly, numerous studies have investigated the prognostic values of DHL or THL, and most of these studies are based on FISH, which is expensive, required expertise and not routinely performed at most institutions. But IHC has the advantages of rapidity, simplicity, economy and high sensitivity, and monoclonal antibodies used in IHC have been gradually commercialized lately, which is suitable to be widespread applied, and has potential ability to be used as a screening method. Secondly, FISH technology is commonly not used to detect genetic deregulation which affects gene expression on the transcriptional and translational levels^19^. But proteins are the functional molecules that play a biological role in the final stage, so protein expression level are likely to represents a more direct measure of the activity of a specific gene. Thirdly, earlier studies using FISH found that no more than 10% of DLBCLs harbored MYC and BCL2 rearrangements. But the recognition of DLBCL with MYC and/or BCL2 overexpression could be used to expand the spectrum of aggressive B-cell lymphomas and effective stratify patients. It can be seen from above that it’s urgent to illuminate the prognostic significance of MYC and/or BCL2 protein overexpression in DLBCL.

MYC oncogene is a transcription factor which play a critical role in cell proliferation, growth, metabolism, differentiation, apoptosis, and immune responses^20^. Its oncology family covering C-MYC, N-MYC, L-MYC that involved in various human cancers. Among them, C-MYC protein overexpression is related to B-cell lymphoma^21^. This gene located on human chromosome arm 8q24, and relevant rearrangements frequently involved other genes. Some evidences show that MYC partner gene is important, and if translocation to non-immunoglobulin (Ig) partner gene, then patients has little or no poor prognosis^22^. A major caused effect of MYC is B-cell proliferation^23,24^. Overexpression MYC has been implicated to play a role in the genesis of numerous human tumors^25^. And overexpression MYC at the protein level as an alternative may be useful to identify cases with inferior outcome because MYC may promotes cellular proliferation through the correlation between protein and serum Vascular Endothelial Growth Factor(s-VEGF)^26^. BCL2 is a central anti-apoptotic gene and located on chromosome 18q21^27^. Approximately 47–58% DLBCLs have BCL2 protein overexpression^28^. The gene controls the apoptosis of body’s normal cells and tumor cells, and involves in physiological DNA repair under normal circumstances. Thus, there is a synergistic effect to accelerate lymphoma progression when both MYC and BCL2 are activated at the same time. That is, MYC promotes cellular proliferation and BCL2 blocks cellular death^29–31^. Chromosomal rearrangements can result in deregulation of MYC and BCL2, but other mechanisms can achieve the same purpose, such as gene gains, amplifications, mutations, activation of nuclear factor-κB (NFκB) pathway signaling^32^ or by micro-RNA-dependent^33,34^ mechanisms. So just checking gene rearrangements by FISH is not enough, all of these reasons mentioned above can cause changes in the amount of protein product finally. As for the relationship between DHL and DE-DLBCL, Green *et al*. shows that the co-overexpression of MYC (implying proliferation) and BCL2 protein (implying anti-apoptosis) is likely a fundamental to the poor prognosis of DHL^35^. But Johnson *et al*. indicated that patients who experience MYC and BCL2 co-overexpression have a poor prognosis regardless of gene translocation^36^. And according to Rosenthal *et al*., the prognosis of DE-DLBCLs is superior than DHLs, the clinical features and outcomes of DE-DLBCLs lies middle between DHL and DLBCL not otherwise specified (NOS). Beyond that, the paper also put forward that DHL is mainly observed in the GCB subset while cases with DE-DLBCL are observed in both ABC and GCB subsets, meanwhile DE-DLBCL encompasses more range than DHL^37^. These two types have close connections as well as many differences. It’s better to carry out prospective multicenter trials in large cohorts of patients that have DE-DLBCL and/or DHL to explain the true correlation between them. The overexpression of MYC and/or BCL2, doesn’t define a new tumor biology but rather, should be considered as an auxiliary prognostic signature that characterized a subset in DLBCL^38^. Aiming at the studies concerning synergistic effect between the two genes, they suggested that the negative prognostic impact of MYC or BCL2 protein overexpression alone was amplified when co-overexpression mixed into them. But there are other reasons can lead to this phenomenon. On the one hand, different studies use different antibodies, fixation methods, staining and scoring methods to perform IHC, and the cut-off values are diverse. On the other hand, we cannot deny the intervention of BCL6 gene or other related mechanisms affecting the results. We expected more studies could explain these complexities by further careful stratify. Given that DLBCL is a highly heterogeneous disease, so it is a good idea to combine other indicators to prediction. These indicators including non-IG MYC partners, BCL6 gene activation and the presence of TP53 mutations or expression, as well as others not listed there. In our study, a single protein overexpression (without considering another protein) has a poor outcome, while the prognosis of co-overexpression is inferior than the two cases mentioned earlier. It also reflects from another aspect that the significance of co-overexpression on prognosis may be more valuable.

Detected the new biomarkers that influenced outcome is absolutely vital in DLBCLs, at the same time, it’s equally important to confirm the standard therapeutic strategies that can play an effective role of improving prognosis in poor risk subgroups. Clinical outcomes of DHL and DE-DLBCL treated with R-CHOP generally exert disappointing, and which has led to recommend intensification of therapy. There have been many studies evaluated the outcomes or complete response (CR) rate treated with different induction regimens in DHL, including dose-adjusted R-EPOCH^39,40^, R-hyper-CVAD^41^, DA-R-ECHOP^42^. But no study demonstrated advantages of using intensive regimen in DE-DLBCLs. The role of autologous stem cell transplant (ASCT) remains controversial in DE-DLBCLs and DHLs with restricted available data and conflicting results. DE-DLBCL was associated with a trend towards reduced progression free survival (PFS) receiving ASCT, but this was not statistically significant due to small sample size^43^. The optimal treatment strategies in these two types is still not well defined as many studies classify them together and outcomes influenced by the incidence of central nervous system (CNS) disease^44,45^. Establishing novel regimens that targeted MYC and/or BCL2 protein illuminates the new direction. BCL2 inhibitors such as ABT-199 may work on BCL2 protein^46^, while developing inhibitors that target MYC protein is not easy owing to its structure.

The current study is the first systematic review and meta-analysis related to the prognosis of MYC and/or BCL2 protein overexpression in R-CHOP treated DLBCLs. Even though, there still remain some deficiencies in the current study. The application of new biomarkers needs certification in a clinical trial or a larger cohort, and a standard therapeutic strategy for such cases remains to be established. Furthermore, relevant analyses of BCL6 weren’t performed due to its rarity. In conclusion, our analysis suggests that MYC and/or BCL2 protein overexpression, and particularly co-overexpression can be readily used to identify patients who have an unsatisfactory prognosis in DLBCL when treated with R-CHOP, and MYC and BCL2 could be candidates for novel therapeutic targets.

### Data availability

All datasets generated and analyzed during the current study are included in this article.
